# A Critical Review of Cannabinoid Metabolites: From Biotransformation to the Urban Water Cycle and Reported Monitoring

**DOI:** 10.3390/toxics14100850

**Published:** 2026-09-24

**Authors:** Abigail Castellanos, Mingjing Sun

**Affiliations:** Department of Chemistry and Physics, Kean University, Union, NJ 07083, USA; castelab@kean.edu

**Keywords:** cannabinoids, cannabinoid metabolites, cytochrome metabolism, water epidemiology, liquid chromatography tandem mass spectrometry (LC-MS/MS), analytical techniques

## Abstract

As cannabis usage persists within communities across regions globally, the aftermath of consumption raises concerns for environmental and public health. The exploration of novel metabolites has expanded knowledge on cannabis metabolism, but the impact of these metabolites when released through bodily excretions into the wastewater system remain limited. Illicit drug consumption can induce adverse effects within the human body in addition to any preexisting health risks and complications; monitoring the release of cannabinoid compounds into the urban water cycle can provide insight into usage patterns and contamination concerns. As legal status of cannabis expands regionally to permit medicinal and recreational uses due to its prospective benefits and psychoactive properties, the rate of consumption and release is expected to increase. Cannabis metabolites within the water system are detected using liquid chromatography paired with tandem mass spectrometry (LC-MS/MS) due to its reliability and specificity. Cannabinoid analysis using LC-MS/MS is cost effective and reduces risk of heat degradation from alternatives like gas chromatography-mass spectrometry (GC-MS). The detection of cannabinoids and their metabolites utilizing primarily LC-MS/MS methodology within past literature is essential for understanding consumption trends and health impacts for residues of tetrahydrocannabinol (THC), cannabinol (CBN), cannabidiol (CBD), cannabigerol (CBG), and cannabichromene (CBC).

## 1. Introduction

Cannabis is categorized into two main species identifications: *Cannabis sativa* (differentiated as hemp) and *Cannabis indica* (differentiated as marijuana), in which cannabinoids are derived from the flowers and leaves but are devoid from the stalks and seeds [1]. Cannabis has over 421 compounds than can be extracted, with a minimum of 113 of these being cannabinoids [2]. Commonly identified cannabinoids throughout research include tetrahydrocannabinol (THC), cannabinol (CBN), cannabidiol (CBD), cannabigerol (CBG), and cannabichromene (CBC) [3]. Historically, cannabis was utilized as food and textile fiber across Europe and Asia, but long-term cultivation and breeding of the plants have led to product development for recreational and pharmaceutical uses [3]. Present-day, cannabis is used to treat a variety of conditions under standardized doses, such as nausea and vomiting, post-traumatic stress disorder, epilepsy, neurodegenerative conditions, anxiety, depression, chronic pains, inflammation, and multiple types of cancer, such as melanoma [4,5]. For recreational use, the aim for consumption varies and is up to the discretion of the user, but intake of THC-heavy products is often accompanied by the risk of side effects like cognitive impairment, addiction, and mental disorders [6].

The cannabis usage is predicted to increase now that accessibility has been expanded legally for 38 U.S. states, Kentucky being the most recent state to pass a bill in 2023 to effectively legalize medicinal cannabis on 1 January 2025 [6]. The Food and Drug Administration (FDA) has classified any products derived from cannabis with 0.3% THC or above as a Schedule I controlled substance, and those at or below 0.3% THC as an agricultural product (hemp) [7,8]. The implementation of the Agricultural Improvement Act (Farm Bill) in 2018 resulted in a broadened availability of extremely pure and concentrated minor cannabinoids federally [7,9,10]. There is a limited understanding of cannabis and its byproducts due to legal restrictions as cannabis is continuously considered a Schedule I drug despite legal expansion [8], prohibiting the direct study of the illicit drug in certain locations and in certain forms. Cannabis is one of the most abused illicit drugs to-date as well, ranking as the third most used controlled substance globally by 2021, following alcohol and tobacco respectively [11]. Legal definitions differ between jurisdictions for cannabis in cultivation, production, and research, creating another layer of limitations [12].

As a result of the developing demand within the cannabis industry, the metabolic pathways through cytochrome enzymes have been addressed with a clearer understanding in recent biological research [13,14,15,16,17,18,19,20,21]. Nonetheless, the identification of metabolites is centrally focused on THC metabolites; this fixation on THC remains a common trend within the analysis of metabolite residues in wastewater to tap water samples [2,22,23,24,25]. This leaves other cannabinoids and metabolites underexplored and a lingering concern due to possible adverse effects. The presence of metabolized cannabinoids among surface and tap/drinking waters can introduce small traces of cannabis to humans and ecosystems that lack prior exposure. For example, THC metabolite 11-nor-9-carboxytetrahydrocannibinol (11-THC-COOH) had been found in concentrations up to 79 ng/L from surface water samples, having a median of 60 ng/L [22,26].

The use of LC-MS in past studies, including methods that are higher in sensitivity for detection (e.g., HPLC-MS/MS), significantly improves the knowledge available in understanding cannabinoids. This method prevents the denaturing of cannabinoids from heating like in gas chromatography analysis and results in more accurate identification of the sample [14]. LC-MS/MS provides a higher specificity for target analytes compared to LC-MS, yet can still result in poor resolution from cannabinoid metabolite structures with extremely similar elution times, such as isomers [27]. Supercritical fluid chromatography offers great selectivity within a shorter time frame, but shifting to this methodology may be a struggle for the analysis of novel cannabinoids due to limited knowledge on retention behaviors within select matrices [27]. Consequently, the presence of various common cannabinoids and their metabolites is often analyzed using LC-MS due to reduced operation costs and maintenance alongside the high sensitivity in detection [28]. Nonetheless, the higher selectivity provided by tandem mass spectrometry (MS/MS) makes it possible for trace levels of cannabinoids to be detected for concentrations as low as ng/L [23], making it a more optimal method for accurate monitoring [28,29].

An evaluation of literature as of May 2026 revealed that while cannabis consumption is an escalating concern, existing reviews lack bridging human metabolism with environmental water systems. The most recent review addressing cannabinoid presence following consumption was published by Kennedy et al. in 2022 [30]. While this work provided vital insights into ecological risks, the scope was largely restricted to Δ9-THC, CBD, and a few metabolites depending on the matrices addressed [30]. Literature published since 2025 targeting cannabis metabolites in general evaluate these compounds within isolated, non-environmental contexts. A review by Sharma et al. focused on how agricultural water deficit stress impacts crop production and plant secondary metabolites, analyzing cultivation concerns only [31]. A study by Mandal et al. reviewed metabolic processes from a forensic and medicinal chemistry perspective involving biological specimens, omitting environmental water matrices [32]. Their emphasis of THC as a predominant target analyte leaves the environmental transport and persistence of other major cannabinoids limited. This review bridges these fields by synthesizing what is currently known about cannabinoid metabolite presence and monitoring, completing the narrative of the urban water cycle from human excretion to wastewater treatment, receiving waters, and ultimately back to human consumption channels.

## 2. Cannabis and Metabolites Within the Body

A variety of cannabis products are available for consumption, such as oils and liquids for vaping, edibles, drinks, concentrates, and topicals [1,25]. The manner of cannabis consumption can vary, such as oral ingestion or inhalation, but will still later be processed within the liver to be either metabolized or excreted [16]. When orally ingested, the cannabinoids reach the liver during the digestion process [16]. When inhaled, the cannabis will first enter the bloodstream through the lungs before travelling to the liver [16]. Cytochrome CYP enzymes present primarily within the liver handle the catalyzation of metabolism reactions for many drugs during Phase-I of metabolism [19]. Cannabinoids can circulate through the bloodstream and are metabolized by enzymes throughout the body during this process [16]. Understanding the impacts of consumption heavily rely on these variables, and are further influenced by factors like liver health, enzyme levels, and concurrent substance usage [5,16,33]. Age, genetics, and sex of an individual are among other factors to consider in future cannabinoid metabolism studies as current literature explores short-term effects of usage [5]. The production of metabolic cannabinoids within the body becomes crucial to understanding their presence and impact once they are released into the environment.

### 2.1. THC, CBN, and CBD Cytochrome Pathways

The mechanisms for THC, CBN, and CBD include a process of hydroxylation, oxidation, and glucuronidation [33]. Phase-I metabolism is the first step which involves oxidation, reduction, hydrolysis, or cyclization to produce active (e.g., 11-OH-THC) and inactive (e.g., 11-THC-COOH) cannabinoid metabolites [33]. Metabolism of THC typically begins when it is ingested and hydroxylated by the liver into 11-hydroxy-Δ9-tetrahydrocannabinol, abbreviated as 11-OH-THC [16]. For this step of hydroxylation, cytochrome CYP2C9 and CYP2C19 mediate the reaction to produce 11-OH-THC as the major pathway [17], which is psychoactive [16]. An alternative cytochrome pathway, CYP3A4, creates minor products 8α-hydroxy-THC, 8β-hydroxy-THC, and 9,10-epoxyhexahydrocannabinol from THC [17,34]. CYP2C9 transforms the 11-OH-THC into 11-COOH-THC by oxidation [17], which is no longer psychoactive [16]. 11-OH-THC reportedly has two minor pathways, one being hydroxylation by CYP3A4 and the other glucuronidation, which produces 8,11-diOH-THC and 11-OH-Gluc-THC, respectively [13,35].

Phase-I metabolism of CBN through human cytochrome P450s results in the production of 11-OH-CBN by CYP2C9 [33,34] and later 11-COOH-CBN [21]. For Phase-I metabolism of CBD, the pathway for hydroxylation and oxidation to form 7-OH-CBD and 7-COOH-CBG includes both CYP2C19 and CYP3A4 [17].

Phase-II metabolism involves conjugation; an article from 2009 determined that the glucuronidation of cannabinoids depended on CYP2C9 and CYP3A4 cytochromes for metabolism of Δ 9-THC, CBN, and CBD [34]. This process serves to deactivate metabolized compounds and increase their water solubility to allow the body to discard these compounds through fecal matter and/or urine [33]. CBD remains untransformed in blood and is ultimately detected as the main product in urine samples [36]. If CBD does undergo any alterations, it is present as the metabolite 7-COOH-CBD in minor quantities within plasma [36]. On the other hand, THC creates several by-products; 11-OH-THC and THC-COOH are the most common [25]. Though studies focus on the legality and biological impact of THC, CBN, and CBD, identifying novel metabolite products creates a baseline in mapping pathways (see Figure 1), beginning with those carried out by cytochrome enzymes.

### 2.2. CBG and CBC Cytochrome Pathways

CBG and CBC are among the lesser studied cannabinoids for metabolites, but the interest in their metabolic pathways has been on the rise due to health benefits and side effect concerns. Analysis of CBG and CBC metabolites has been increasingly gaining interest predominantly in how exactly its metabolic pathway through cytochromes impacts the human body. While CBG lacks any psychoactive effects, consumption has increased in part to its positive impacts for relieving pain, cancer, asthma, and arthritis symptoms [19]. Similarly to CBG, CBC is nonpsychoactive and diverse in treatment applications for the central nervous system regarding injury, damage, or inflammation [19]. CBC analysis is limited because of its minimal presence in cannabis, making the process difficult in knowing its chemical properties and interactions with other compounds [19].

Any existing studies that explore the metabolic pathway of CBG were published around the early 1990s; it has been generally established that CBG follows the same major pathway as THC, CBN, and CBD for hydroxylation, then oxidation, and finally glucuronidation [33]. A 2022 study researching the metabolism of CBG by cytochrome P450s determines a definite major and minor pathway, in which the major product is cyclo-CBG following cyclization and the minor product is 6′,7′-epoxy-CBG following epoxidation (see Figure 2) [18]. When testing all CYP cytochromes, cyclo-CBG formed approximately 100 times more often than 6′,7′-epoxy-CBG. This pattern was reinforced by the quantification of cyclo-CBG and 6′,7′-epoxy-CBG using mass spectrometry, where the amount produced was 100 times higher for cyclo-CBG than 6′,7′-epoxy-CBG [18]. Both CBG metabolites were newly reported through this study were found to decrease inflammation and serve as an antioxidant when tested on animals [18].

CBC metabolic pathways has been a growing point of interest as studies have determined a higher absorption of CBC in plasma than that of THC and CBG in the human body when the three cannabinoids are consumed simultaneously [19]. Once CBC is within the human liver, cytochrome enzymes mediate the production of CBC metabolites similarly to other cannabinoid metabolism trends [19]. Studies addressing metabolism of CBC through animal testing have elucidated an epoxidation reaction taking place regardless of the type of animal, with only the quantity of metabolites produced depending on animal type [20]. The 2024 study by Roy et al. explored the specific cytochrome P450 enzymes that helped to map the metabolic routes of 8′hydroxy-Cannabichromene, 6′,7′-epoxycannabichromene, and 1″-hydroxy-cannabichromene (see Figure 2). In a study by Ward et al. utilizing human liver microsomes, a major metabolite of CBC was identified as 2′-hydroxycannabitcitran (2′-OH-CBT). Unfortunately, there is a severe lack of studies that dive into the metabolites, identification of produced metabolites, and the effects of these drug residues once outside of the body.

## 3. Cannabis from the Body to the Environment

The connection of cannabinoid metabolites to environmental and public health has been addressed through water treatment system studies and their success in removing drug residues [22,36,37]. The extent of cannabinoids being cycled through this water system is underexplored; many existing studies containing data with a profound focus on THC and THC-COOH more than any other cannabinoids (see Figure 3). The studies on THC and THC-COOH provide a foundation for the detection and analysis of other cannabinoid compounds as environmental and health concerns increase.

### 3.1. Human Consumption to Sewage

Metabolism within the human body creates cannabinoid byproducts that remain understudied, even for common cannabinoids like CBN, CBD, CBG, and CBC [40]. THC derivatives remain recognized as the most common cannabinoids, primarily in the forms of 11-OH-THC and 11-COOH-THC [30]. THC exists in smaller quantities within bodily excretions compared to THC-COOH as it undergoes metabolism after consumption [36]. The presence of major THC metabolites depends on the type of bodily waste excreted, such as 11-COOH-THC being present mainly in urine as a glucuronide conjugate while 11-OH-THC is greatest in fecal matter [16]. As for CBD, 7-COOH-CBD is the most common metabolite that is excreted, either in its original form or as a glucuronide [16]. Over 65% of cannabis is released in fecal waste with about 20% released through urination, taking approximately 5 days to excrete the majority (reported as 80–90%) of consumed cannabis as metabolites [16].

Urine analysis has demonstrated that metabolite THC-COOH stays mostly intact when entering the sewage system, no degradation of the compound being present at this stage of release for facilities that use chlorination as a pre-treatment [41]. When urine samples were diluted to contain only a tenth of urine and spiked with chlorine (1 mg/L), the concentration of THC-COOH continued to be relatively the same [41]. Though this does not account for other types of bodily waste containing cannabis metabolites, it confirms cannabis metabolites being released into the water system in at least one form of excretion. Influent sewage water analyzed by Postigo et al. contained THC, OH-THC, and THC-COOH at concentrations of 48.4 ng/L, 2.3–90.9 ng/L, and 10.6–21.7 ng/L, respectively [42]. On a college campus in Florida, THC-COOH was detected in raw sewage samples at a range of 30–2413 ng/L [43].

Within sewage sludge samples, the cannabis markers were greater than in water samples; this was attributed to the lipophilic nature of cannabinoids [44]. Nonetheless, it is crucial to note that whenever sludge containing THC is surrounded by water with a lower concentration of THC, they are released into the water [2]. THC was detected at a range of 29.9–579.0 ng/g d.w. ng per gram of dry weight of sludge) in 100% of sewage sludge samples collected from 15 different wastewater treatment plants across Spain [45]. OH-THC was present at a detection frequency of 67% with a concentration range of 10.3–160.0 ng/g d.w. In this study, the detection of CBN and CBD within samples is the first published evidence of their presence within sewage sludge; their concentration ranges were 26.7–188.0 ng/g d.w. and 32.2–489.0 ng/g d.w., respectively [45]. CBN had a detection frequency of 53%, the lowest out of the cannabinoids tested [45]. CBD was the second most frequent cannabinoid detected at 80%, following THC [45].

Sludge sample investigations for cannabinoid residues are minimal, most studies focus on activated sludge (sludge undergoing secondary treatment by wastewater plants) [44,46]. To tackle this, Pandopulos et al. developed a sensitive method for the detection of 50 cannabinoids in sludge samples gathered from a major municipal treatment plant located in Australia [44]. It was reported that THC (3200 µg/kg), THC-COOH (1180 µg/kg), and CBD (127 µg/kg) were the highest concentrations found within primary sludge [44]. Other metabolites were tested as well, such as 7-OH-CBD and 7-COOH-CBD, but concentrations were not explicitly disclosed [44]. Nonetheless, sewage sludge can reveal transformation trends and effectiveness of removal as it enters wastewater treatment plants [44,45].

### 3.2. Wastewater Treatment Plant Process

Wastewater treatment plants are often not equipped with the tools to remove common drugs, including THC and THC metabolites despite the dominating scientific focus on it as a major cannabinoid [47]. González-Mariño et al. found that the treatment of these residues through water chlorination leaves by-products that are more toxic than the original metabolite [41]. Traditional wastewater plants are constructed to handle the removal of pollutants like organic matter particles, pathogens, and suspended debris, but are not effective in removing pharmaceutical or personal care product residues [37]. The efficiency of cannabis residue removal for within wastewater plants is often calculated as <50% [41], but ranges can vary greatly from −18.3% to 100% in some cases [23]. Hydrolysis of THC-COOH conjugates can lead to a larger presence of THC-COOH within effluent water samples, which can imply a trend of post-treatment quantification of residues being higher than that of influent samples for some facilities [41].

Primary treatment and secondary treatment of water is often conducted as removal of sediments and aeration to further break down particles [36], but can go up to tertiary treatments that often consists of ozonation, ultraviolet photodegradation, or chlorination [39]. These differing conditions can lead to variability in cannabinoid concentrations, even if the sample is collected within the same step of the wastewater treatment plant [48]. This variability stands true regardless of the type of sample that is collected, specifically observed in both suspended debris and influent wastewater [48]. Within the study by Boix et al., THC-COOH was detected in all the wastewater samples analyzed regardless of influent or effluent status [39].

Wastewater treatments can result in transformation products of cannabinoids and their metabolites [39]. For hydrolysis, wastewater is kept in dark environment at room temperature to allow reaction to carry out [39]. About 20% of THC-COOH later degraded into C_20_H_27_O_4_, which has a mass of 331.1909 (*m*/*z*); the highest concentration of this degradation product was seen after 10 days [39]. For photodegradation, UV lamps are used to treat wastewater; 254 nm wavelength or broad spectrum (200–400 nm) break apart particles [49]. THC-COOH degraded after 30 min of exposure and produced three transformation products that disappeared in about 4 h, demonstrating that UV is effective [mercury lamp at 254 nm used] [39]. For chlorination, usually NaClO, HOCl or ClO is added to wastewater for disinfection [39]. THC-COOH degrades quickly (in about 5 min when 40 µL 1% NaClO was added to 50 mL of spiked sample); eight different transformation products were detected after [39].

THC-COOH has been detected in wastewater with concentrations as high as 2591 ng/L for untreated water and 753 ng/L following treatment [36]. Boix et al. reported a mean concentration of THC-COOH in influent and effluent wastewater collected in Spain as 56 ng/L and 8 ng/L, respectively [39]. Tested samples in a Dutch wastewater study had a detection frequency of 88% for THC-COOH in influent samples and 9% for effluent samples [50]. After having tested wastewater samples from a Northern Ireland Prison, there was no successful detection of THC-COOH as a possible result of dilution within the water; similar research establishes low frequency as an issue for detection [51]. However, surface waters from remote Ireland lakes that were tested for compounds of wastewater origin contained THC-COOH at an overall range of 12 to 76 ng/L from four different locations [52]. Cannabinoid metabolites are escaping into surface waters from wastewater treatment plants with key information narrowed to THC and THC-COOH.

### 3.3. Surface Waters

The partial removal of metabolites leads to their presence in surface water. THC-COOH has the greatest longevity once in the environment compared to other cannabinoids, making it one of the best markers for the analysis of cannabis consumption trends within a population [25,39]. THC-COOH was detected in surface waters in Italy (0.3–3.7 ng/L), Switzerland (0.3–4.9 ng/L), United Kingdom (0.6 ng/L), Spain (3.4–79.7 ng/L), and the United States (15 ng/L) [36]. In a study utilizing samples from Spain, surface waters contained THC-COOH and OH-THC at 5.5 ng/L and 0.4 ng/L, respectively [42]. Additionally, surface water from the Tagus River in Spain contained a concentration of 6.51 ng/L for THC-COOH within one of the five tested samples, but lacked any traces of OH-THC [38].

The trace level concentrations of OH-THC within previous studies has been attributed to the metabolism of OH-THC to THC-COOH within the human body [38] and its affinity to separate into solid waste once in the aquatic cycle [24]. Few address the impacts of these cannabinoids being present in water systems (see Table 1), but existing research has demonstrated that the toxicity of THC-COOH on zebra mussels has led to increased DNA fragmentation with no distinct genetic damage after 14 days of exposure to 500 ng/L of THC [53]. Another report observed increased anxiety behaviors in zebrafish when exposed to THC concentrations higher than 30 mg/L [54]. The effects of metabolites on environmental and human health are not well-known despite findings indicating that higher availability leading to an increased likelihood of health concerns [55]. Bioaccumulation of these residues is predicted to be likely due to the lipophilic nature of cannabinoids [30]. In spite of this potential trend, no direct correlation between cannabinoid pollution and health has been made beyond the long-term exposure of cannabinoids like THC and CBD in lab-settings utilizing animal models [56]. Impacts of cannabinoids in the environment heavily relies on bioavailability, and though trace levels of cannabinoids within surface waters do not pose any fatal hazards, the behavioral and genetic changes from exposure in past studies should not be overlooked [30,56,57].

### 3.4. Drinking Water Treatment Plants and Re-Consumption of Residues Through Tap

Many publications fail to address the re-consumption of cannabinoids and their metabolites after these residues reach tap water, often considered safe for drinking depending on region. THC-COOH has been reportedly detected at a concentration of 1 ng/L for tap water when described by a 2017 literature review [23], yet an inconsistency in this observation was noted upon checking the original source for this citation. The analysis of tap water samples cited in this review had been conducted in 2011; although illicit drugs were reported to be present in levels less than 1 ng/L, the original researchers clarified that THC, THC-OH, and THC-COOH were not actually detected in any of the seventy tap water samples within the abstract [59]. The 2011 study reported detections of THC and its metabolites in wastewater and surface water, however, ascertaining the chlorination process of treatment plants as the reason cannabinoids are not later detected in tap water collected across countries (categorized as Spain, European countries, Japan, and Latin-American countries) [59].

There is limited scientific investigation on whether any metabolites have been detected in treated drinking water [2,60]. When tested, THC, OH-THC, THC-COOH, CBN, and CBD were not observed within any of the samples for drinking water across central Spain [60]. However, in a review of the occurrence of THC and metabolites, it was listed that THC-COOH was present in raw drinking water up to a concentration of 14.7 ng/L [2,26] and in treated drinking water up to 1 ng/L [2,46]. OH-THC was not listed within this report [2], but a 2012 study reported the detection of OH-THC at 0.49 ng/L [24,38]. A more recent study conducted in 2022 by Muñiz-Bustamante et al. on illicit drug consumption in Europe failed to detect cannabinoids in any of the 119 tap water samples collected [61]. These low traces of metabolites are attributed to degradation, effective water treatment like chlorination, and the absorption of these cannabinoids to solids throughout the process [24].

Minor occurrences of metabolites being reintroduced to the human body through tap water entails cannabis metabolite absorption by those who have never consumed cannabis directly. It is important to emphasize that metabolites that have been detected in the past are limited, especially in terms of human re-consumption, but current data implicates little to no risk to human health at trace levels [24]. Still, cannabinoid detection for tap waters remains a necessity as toxicity of transformation products and effects of concurrent exposure with other pollutants are generally unknown [61]. Due to its classification as a drug of abuse and broadened accessibility, cannabis residues at this stage of the urban water cycle can provide further insights into accumulation patterns and efficiency of drinking water facility treatments [56]. Additionally, long-term drinking water cannabinoid contamination may influence the well-being of vulnerable groups compared to the average healthy adult human, even at trace levels. The quantities of cannabis released by communities can become difficult to measure in samples due to degradation, legal availability, and environmental factors (e.g., temperature, precipitation, radiation) [37]. Without full comprehension of cannabinoids from the body to the water system, chemical and physical properties are ambiguous.

## 4. Analytical Method for Targeted Samples

Analytical method references cited were found using the databases Google Scholar, ScienceDirect, and EBSCO Research. Selection criteria for the articles referenced were the inclusion of keywords: “cannabinoids,” “’cannabinoids’ and ‘[water/biological] analysis using [LC-MS/GC-MS/SFC],” “detection of cannabinoids using [LC-MS/GC-MS/SFC],” “’cannabinoid metabolites’ and ‘[LC-MS/GC-MS/SFC] analysis’,” and “cannabis metabolites.” If available, filters like Peer Reviewed and Year were selected. Articles for LC-MS methods for biological samples within the past 5 years were selected, excluding the study by Cruz-Espindola in 2018. For water epidemiology studies involving wastewater to surface waters, articles published after 2008 were utilized, the oldest reference being from 2009. Articles were selected based on successful quantification of any target cannabinoids. For tap water analysis, no time restraint was implemented due to limited availability of data; methods that were successful in detection were chosen and categorized using “DW” (drinking water). No literature was excluded other than for the time restrictions mentioned. Many articles were selected from citations, such as articles from before 2010, due to prevalence in research to bridge information collected regarding water epidemiology.

### 4.1. Biological Samples Treatment and Analysis

The identification of metabolites within biological samples is crucial to understanding their transformations within the body and their chemical properties. To analyze the types of metabolites present, biological samples are taken from individuals who consumed cannabis, which can be useful to identify metabolic structures previously unstudied [62] and are insightful to health impacts on organisms [28]. Types of biological samples prominent within recent metabolomic research include whole blood, blood plasma, urine, and hair for drug testing. However, other matrices that can be taken for analysis of cannabinoids are nails, serum, oral fluid, and even exhaled breaths [28]. Multiple methods have been developed to analyze chemical differences between matrices to detect cannabinoids, target compounds often being THC, CBN, CBD, CBG, and CBC, along with their metabolite derivatives [4].

The most common method of detection tends to be a combination of tandem mass spectrometry (MS/MS) with gas chromatography (GC) or liquid chromatography (LC) [4,28]. Liquid chromatography is often the preferred route due to its shorter run time, high selectivity, and less costly preparation compared to GC [4,28]. For GC, the samples undergo high temperatures within the column, leading to the decarboxylation of acidic cannabinoids during the process if the compound is not derivatized beforehand [28]. The need to derivatize samples for GC analysis is eliminated for the detection of cannabinoids when utilizing LC, posing less complications in sample testing as a result [28,63]. With these factors, the HPLC method is generally considered the gold standard for cannabinoid identification and detection as it is versatile and sensitive [64]. It is important to note that GC analysis is an incredible method for detection within complex matrices like blood plasma, finding that sensitivity of target cannabinoid detection can be increased through optimizing derivatization prior [65]. Supercritical fluid chromatography is also renowned for the separation of cannabinoids in complex matrices by implementing gases like CO_2_ [66]. This type of analysis has grown in usage due to carbon dioxide’s status as an environmentally friendly solvent and nontoxicity while being highly selective as a mobile phase for target cannabinoids within samples [66]. Depending on the goal of analysis and analytes, LC for biological samples may result in heightened use of organic solvents, but this method provides the room for the analysis of both neutral and acidic cannabinoids without risking sensitivity during initial sample preparation [64].

Sample preparation is often conducted using solid-phase extraction (SPE) based on sample type and quantity [63]. SPE has actively provided the best recovery rate, but recent research expands to using other minor extraction methods to reduce time and material usage for analysis [63]. These other methodologies for sample preparation of biological samples are often solid-liquid extraction (SLE) for solids like hair and nails; QuEChERS for plasma, blood, and urine; and dispersive liquid-liquid microextraction (DLLME) for urine (see Table 2) [28].

### 4.2. Water System Samples: Wastewater to Drinking Water

#### 4.2.1. Water Sample Treatment

Sample treatment and storage can impact the reliability of detection as some conditions may be better suited for specific sample types when analyzing cannabinoids. Cannabinoid metabolite loss can occur simply from the type of container the sample is kept in due to adsorption [2]. When using deionized water and urine samples spiked with THC-COOH, untreated amber glass containers resulted in the lowest metabolite loss when kept at a temperature range of 2–8 °C within a 16-h period [2]. Other losses can originate from procedural factors, such as pipette material and solvents used [2]. Stability of samples in storage depends on sample type and solvents used to prevent long-term microbial growth [2,76]. Different storage methods are specific to the analyte(s) later detected using LC-MS (see Table 3).

#### 4.2.2. Water Sample Analysis

LC-MS conditions are highly specific to the analyte and can be useful when the targeted compound is present at trace levels. This review focuses on LC-MS methodology due to the degradation of cannabinoids that result from gas chromatography (GC) from heating, making LC a preferable means to analyze water samples [14]. Additionally, LC analysis is useful due to its speed and specificity to assess the degree of drug consumption [40]. The conditions of LC-MS analysis used for illicit drug in water sample research (Table 4) correspond to the sample treatment outline in the previous section (Table 3).

Gas chromatography is predominant within biological sample analysis yet limited within water epidemiology for cannabinoids [2,47,78]. THC-COOH was detected at 7000 ng/L in influent waters [47], using SPE with HLB cartridges for sample treatment method prior to GC-MS [47]. This was the lowest concentration THC-COOH detected in the study when compared to other concerning contaminants like amphetamine, methamphetamine, and benzoylecgonine [47]. Within raw wastewater, THC and THC-COOH were detected at ranges 12–35 ng/L and 50–153 ng/L, respectively [78]. The recovery ranged from 94–104% for THC and 92–112% for THC-COOH when using GC-MS as an analytical method for raw wastewater, treated wastewater, and surface water samples [78].

Supercritical fluid chromatography (SFC) gained traction as a fast analytical method for the detection of cannabinoids, especially for methods utilizing a UHPSFC system [79]. SFC is predominantly an analytical method of cannabis products, such as edibles, rather than water matrices’ cannabis residues [80,81]. The only study cited for SFC wastewater analysis is by González-Mariño et al., where ultra-high performance SFC is paired with tandem mass spectrometry (UHPSFC-MS/MS) [82]. This was the first instance of implementing UHPSFC-MS/MS to detect cannabinoids in wastewater, resulting in an LOQ range of 1–59 ng/L [82,83]. In 2023, a study monitoring polar organic micropollutants in effluent wastewater found a transformation product likely originating from synthetic cannabinoids, identified as 17α-hydroxypregenolone [84]. This was the last known instance of SFC analysis to wastewater samples in detecting cannabinoids.

## 5. Discussion: Limitations and Future Approach

The subject of cannabinoids and their metabolites have progressed in biological contexts from recognition of novel metabolic products, analysis in different sample matrices, and pathway exploration of cannabinoids. The presence of cannabinoids in biological studies has readily available research as recent as 2022–2024 on metabolites [28] like THC, 11-OH-THC, 11-COOH-THC, 8, 11-diOH-∆9-THC, 11-COOH-THC-Gluc, CBN, CBD, 7-OH-CBD, 7-COOH-CBD, CBG, and CBC. However, water system sample findings were heavily restricted to THC and THC-COOH, failing to address common metabolites like CBG or CBC altogether. In some cases, recent studies were the first instance of research on specific cannabinoids or the identification of them; Milan et al. reported that CBDA, CBDV, CBG, and THCA were newly considered cannabinoids within their study of waste and river water to their knowledge. Additionally, a study by Ward et al. noted the identification of CBC metabolites produced by cytochrome P450s by Roy et al. but pointed out that novel metabolites (like 2′-hydroxycannabicitran) were unaddressed. Water-based epidemiology for metabolite presence needs to be on-par with biological advances in determining the impact of residues on environmental and public health.

The presence of various cannabinoid metabolites can currently indicate the consumption trends of cannabis. For example, THC-COOH often has the longest window for detection, its structure being the most stable in urine and blood [85]. On the other hand, the identification of metabolic compounds in a variety of matrices can determine the chemical behaviors and risks involved with its interactions biologically and/or environmentally. This can be phenomenal for metabolites that underwent Phase II metabolism, but a thorough comprehension of their bonding affinity continues to be limited even within the human body [35]. Cannabinoid receptor binding and effects of each metabolite require additional analysis as metabolic pathways are gradually identified [13,15,18,19,33]. Potential activity of certain metabolites to CB1 and CB2 receptors were sometimes unclear [20] or unexplored; these were also unaddressed within this review. Identifying biological and chemical properties of metabolites can provide a beginning point in analyzing their long-term environmental effects. Despite the detection of cannabinoids within samples at each point of the water system and biosolid samples taken from the system [44], there is no study on the introduction or re-consumption of cannabinoids from surface or tap waters to organisms who have never consumed cannabis directly [30].

The most successful method for detection and quantification within biological sample and urban water matrices was LC-MS/MS (see Table 2 and Table 4). While sample preparation can vary greatly from SPE to LLE depending on the sample type, SPE has been implemented in water-epidemiology the greatest due to higher optimization of analysis on analyte concentrations present [45]. LLE is advantageous due to easy repeatability while providing a great recovery of cannabinoids, but requires a large volume of sample and solvents, which can be expensive [28]. Arguably, SPE can be costly as the cartridges used for extraction can only be used once and may require large volumes of sample as well; the benefits of automation and reduction in interference are what ultimately set apart SPE from other preparation methods [28,61]. Other sensitive means of analysis like GC-MS methods are often not ideal for cannabinoid studies as a result of their acidic nature, high operating temperatures risking the decarboxylation of many structures regardless of the original sample matrix without additional steps to prevent it [12]. LC-MS/MS is preeminent across sample matrices for cannabinoid analytes for its reduced operation time, high resolution, and simpler pretreatment processes [28,45,61].

## 6. Conclusions

Despite any existing limitations within the determination of cannabis and their metabolites on environmental and public health, expanding current knowledge through sensitive analysis can bridge the gaps between existing literature. LC-MS remains the preferred analytical method for water epidemiology, but other techniques like GC and SFC continue to be developed and implemented for the detection of cannabinoids accordingly. As the cannabis industry is ever-growing with a variety of manners for consumption, current research has determined a profound need for further exploration of residues beyond THC and THC-COOH. The potential of cannabinoid metabolite bioaccumulating is not certain as both wastewater treatment plants and drinking water plants can vary in removal efficiency depending on treatments enforced. This uncertainty presents the possibility of exposure to cannabinoid metabolites, even at trace levels, as THC metabolites specifically have been successfully detected at least once at every point of the water system passage explored. The investigation of cannabinoid metabolites from the human body to our water system can be a push towards accurate estimates on environmental and public health when navigating adverse effects of cannabis consumption growth.

## Figures and Tables

**Figure 1:**
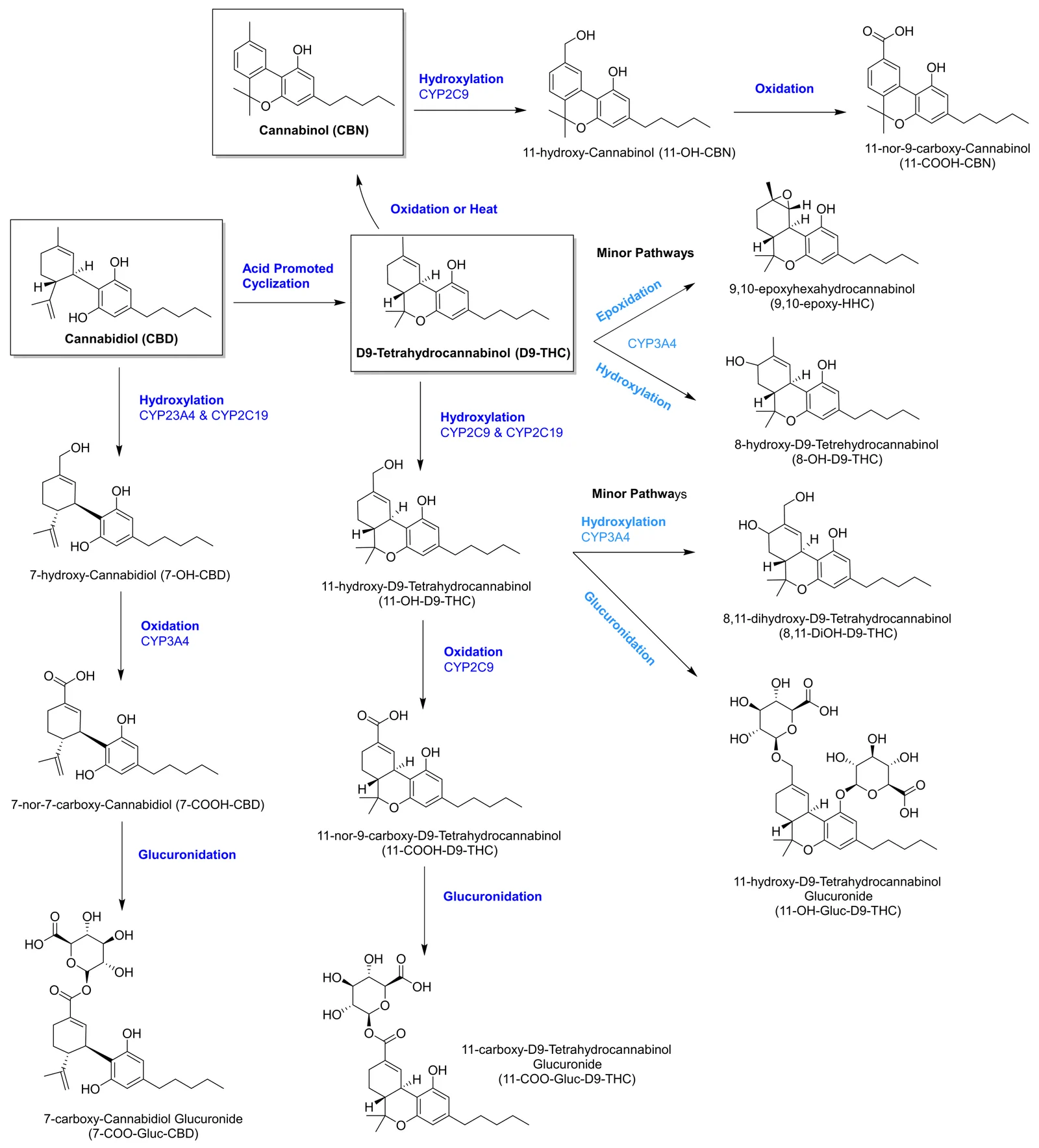
Flowchart of Metabolic Pathways for Common Cannabinoids Δ9-THC, CBN, and CBD [13,14,17,34]. Minor pathways are labelled accordingly and in a lighter blue; all other pathways are in a dark blue. If available, the CYP P450 enzymes responsible for the metabolism of a specific compound are provided.

**Figure 2:**
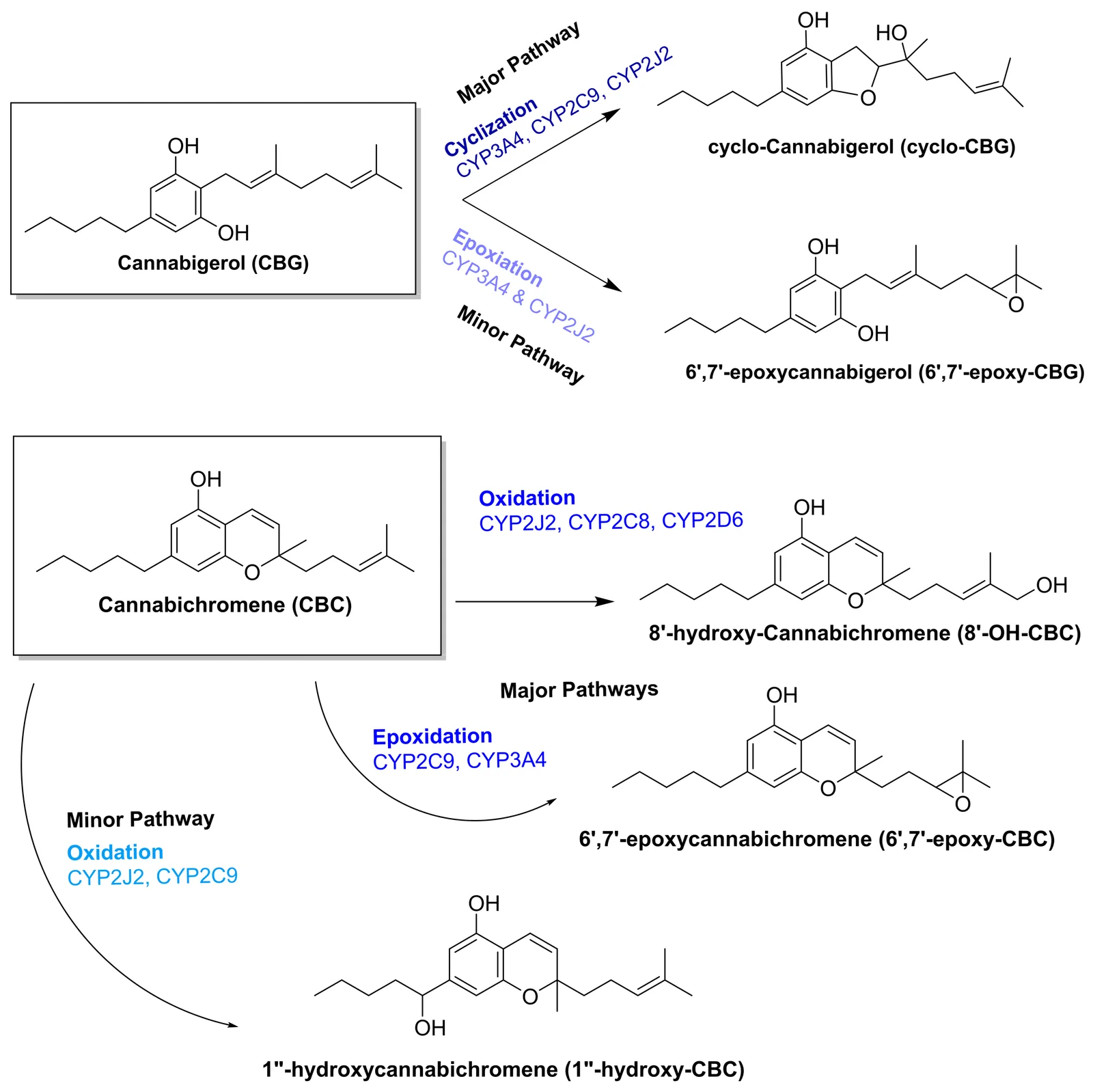
Flowchart of Metabolic Pathways through Cytochrome P450 Enzymes for Common Cannabinoid CBG and CBC [18,19]. For each common cannabinoid, major and minor pathways are labelled. Minor pathway information is in a lighter shade of color than the major pathway for visual reference, such as the CBC major pathways displayed in dark blue with the minor pathway being shown in a light blue. The reaction the parent compound undergoes in the presence of specific CYP P450 enzymes is described accordingly.

**Figure 3:**
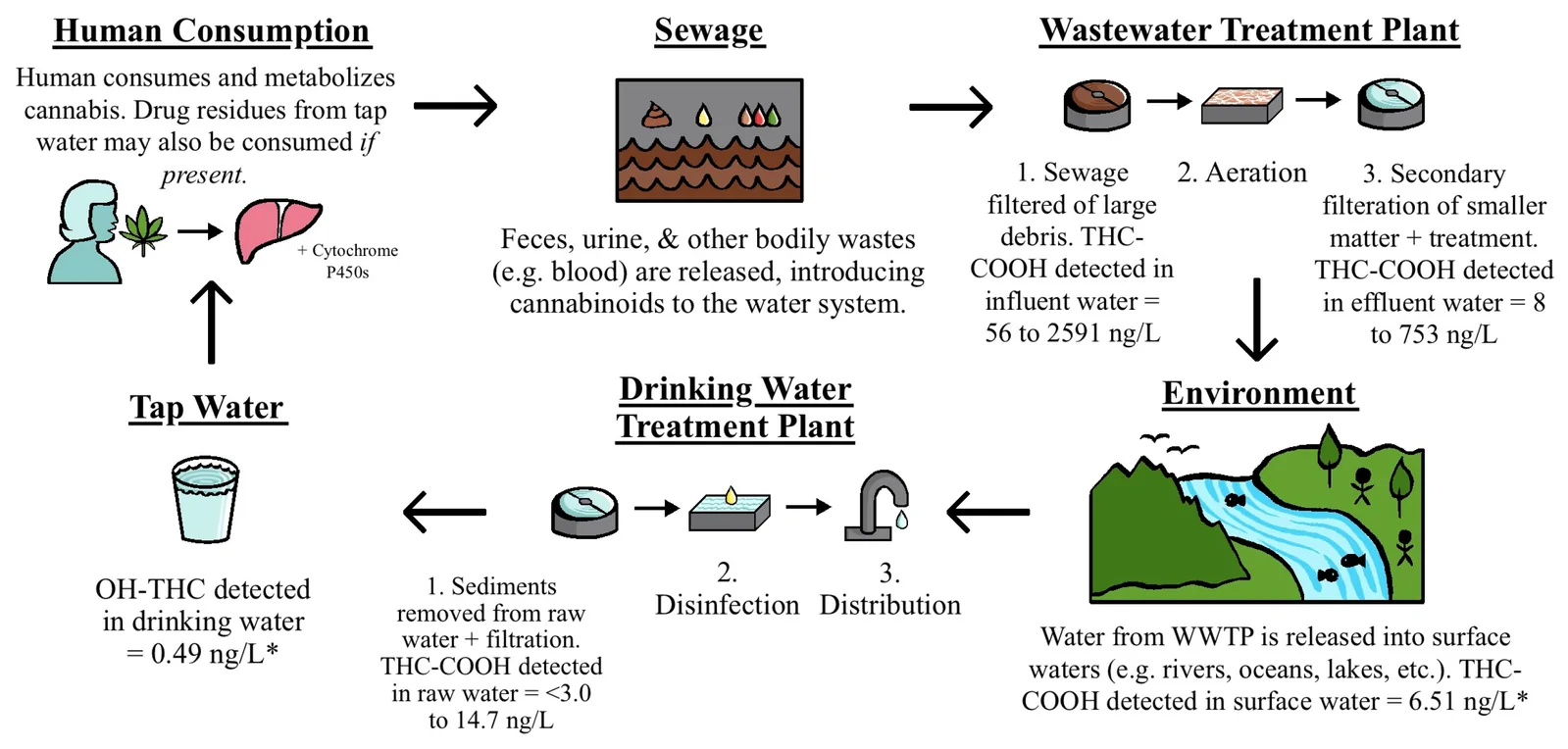
General flowchart of cannabis and metabolites traveling from the body through the water system [26,36,38,39]. * Concentration from Valcárcel et al., 2012 [38] specifically.

**Table 1:** Overview of THC, CBN, CBD, CBG, and CBC Detection in Effluent Wastewater and Surface Water.

Cannabinoid	Detected Concentration: Effluent Water	Detected Concentration: Surface Water	Key Information	References
Tetrahydrocannabinol (THC)	20.5 ng/L *	13.6 ng/L *	(1) THC-COOH and 11-OH-THC have been detected in surface waters. (2) Cannabinoid presence spans across Europe, the U.S., Costa Rica, Colombia, and Martinique.	(1) [42] (2) [56] * [2]
Cannabinol (CBN)	0.076 µg/L *	0.079 µg/L *	(1) CBN has been detected in wastewater, groundwater (urban areas), and in airborne particles.	(1) [40] * [40]
Cannabidiol (CBD)	0.096 µg/L *	<LOQ, LOQ = 6.2 (10 µg/L) *	(1) Chlorine can react with CBD’s double bonds that are not a part of its phenol ring structure in an oxidation and halogenation reaction. (2) CBD concentrations were found to be lower than that of THC within effluent samples than in raw sewage samples, still risking being released into surface waters following treatment. (3) Like CBN, CBD has been found in groundwater and airborne particles as well.	(1) [36] (2) [56] (3) [40] * [40]
Cannabigerol (CBG)	<LOQ, LOQ = 2.1 (10 µg/L) *	<LOQ, LOQ = 2.1 (10 µg/L) *	CBG has not been considered in a previous wastewater study and has not been considered in any recent surface water studies beyond the starred source for this row at the time of this review; no further information can be provided regarding CBG at this time.	* [40]
Cannabichromene (CBC)	No known wastewater analysis studies.	No known surface water studies.	No known wastewater or surface water studies mention CBC at the time of this review.	None available.
Consequences on Wildlife within Exposed Environments				References
(1) Exposure to THC and THC-COOH residues resulted in DNA fragmentation, with oxidative damage observed only in higher concentrations (1000 ng/L). (2) Cannabis exposure in general demonstrates physiological and behavioral impacts among carp, tilapia, and zebrafish; mortality rates for mosquitos and snails increased. (3) Embryo development of zebrafish when exposed to THC (6 mg/L) and CBD (3 mg/L) indicated changes to neuron and muscle morphology, but it is noted that these concentrations don’t occur naturally in surface waters. Nonetheless, it can demonstrate potential negative impacts.				(1) [57] (2) [56] (3) [2,58]

* Data Sources.

**Table 2:** Sample Treatment and LC-MS Method Outline for Biological Samples.

Analyte	Matrix	Sample Treatment	LC-MS Method	(LOD/LOQ)	Ref.
CBG THC	Whole blood (Human)	SPE (Bond Elut Plexa SPE Cartridges 6 mL, 200 mg—Conditioned with 2 mL MeOH and 2 mL DI water)	UPLC-MS/MS (Agilent 1290 UPLC System—Agilent 6460 Triple Quad MS—Agilent Jet Stream ESI Source; Restek Raptor Biphenyl Column, 2.1 mm × 100 mm × 5 µm) Agilent Technologies. Santa Clara, California, United States (A: 5 mM ammonium acetate/0.1% acetic acid, B:15% MeOH in ACN)	(0.2/0.5) µg/L	[67,68]
CBD 7-OH-CBD 7-COOH-CBD	Blood plasma (Human)	SPP (Phree Phospholipid Removal (PLR) 96-well plates—Treated with 300 µL ACN and IS)	UHPLC-MS/MS (Agilent 1290 Infinity II UHPLC System—OptiFlow Pro Ion Source; Acquity UPLC BEH Phenyl Column, 2.1 mm × 50 mm × 1.7 µm) Agilent Technologies. Santa Clara, California, United States (A: 5 mM ammonium carbonate, B: 100% ACN)	(No LOD/ 1.95–31.25) ng/mL	[69]
CBN THC (Δ8/Δ9) 11-OH-THC 11-COOH-THC THC-COOH-Gluc THCA CBD 7-OH-CBD CBDA	Blood plasma (Human)	10 µL of IS added to 100 µL of sample; analyte extraction done by adding 300 µL of frozen-ACN (−20 °C)	UPLC-MS/MS (Acquity H-Class PLUS Waters—Xevo TQ-S Waters MS/MS; Kinetex Polar C18 Column, 2.1 mm × 100 mm × 2.6 µm) Waters Corporation. Milford, Massachusetts, United States (A: 0.1 *v*/*v* formic acid in water, B: 0.1% formic acid in ACN)	(0.15–0.87/5) ng/mL	[70]
CBD 6α/β-OH-CBD 7-OH-CBD 7-COOH-CBD CBD-Gluc THC 11-OH-THC THC-COOH THC-COOH-Gluc THC-Gluc CBC CBN CBG THCV CBDV THCV-COOH	Blood plasma (Human)	Each 200 µL aliquots of plasma sample spiked; 800 µL of 30% water with 0.2 M ZnSO_4_/70% MeOH and IS added.	HPLC-MS/MS (Agilent 1260 System—AB Sciex API5500 Triple Quad MS with Turbo V Ion Spray; Ascentis Express RP-Amide Analytical Column, 3.0 mm × 150 mm × 2.7 µm) Agilent Technologies. Santa Clara, California, United States (A: 0.04% formic acid in water, B: ACN/MeOH/Isopropanol (3:1:1 *v*/*v*/*v*))	(No LOD/ 0.78–7.8) ng/mL	[4]
CBD CBC CBG CBN THC	Blood plasma (Canine)	SPE (Oasis HLB Cartridges—Conditioned with 2 mL MeOH and 2.0 mL distilled water; eluted with 1 mL MeOH)	UHPLC-MS/MS (Agilent 1290 UPLC System—Agilent 6460 Triple Quad MS with Jet Stream ESI; Agilent ZORBAX Eclipse Plus C18 Column, 2.1 mm × 50 mm × 1.8 µm) Agilent Technologies. Santa Clara, California, United States (A: 0.1% formaic acid in water, B: ACN)	(1.95/3.91–15.63) ng/mL	[29]
THC OH-THC THC-COOH CBD CBN	Urine (Human)	LLE & SLE (Agilent Chem Elut 96-well plate 400 µL)	LC-MS/MS (Agilent 1290 Infinity II LC System—Agilent 6490 Triple Quad MS; Agilent ZORBAX Eclipse C18 Column, 2.1 mm × 100 mm × 1.8 µm) Agilent Technologies. Santa Clara, California, United States (A: 10 mM ammonium formate, 0.5 mM ammonium fluoride in water, 0.125% formic acid, B: 10 mM ammonium formate, 0.5 mM ammonium fluoride in 95:5 ACN/water)	(No LOD/2) ng/mL	[71]
CBD-COOH THCV-COOH 11-OH-THC THC-COOH CBG THCV CBD CBN THC	Urine (Human)	Hydrolysis & SPE (10 mL Styre Screen THC, 60 mg, SPE columns—Conditioned with 1 mL MeOH and 1 mL DI water. Eluted with 3 mL of hexane/ethyl acetate/glacial acetic acid 49:49:2 *v*/*v*)	LC-MS/MS (Shimadzu Nexera LC—Sciex 3200 API MS; Agilent Poroshell 120 EC-C18 Column, 2.1 mm × 100 mm × 2.7 µm) Shimadzu Corporation. Kyoto, Japan (A: 0.1% formic acid in ultrapure water, B: 0.1% formic acid in ACN)	(1–5/No LOQ) µg/L	[72]
THC (Δ9)/THC Isomers 11-OH-THC (Δ8/9) 11-COOH-THC (Δ8/9) Δ8-THCO-Acetate	Urine (Human)	100 µL of β-glucuronidase added to every 50 µL aliquot of urine. 25 ng IS and 325 µL water/MeOH (1:1) added.	HPLC-MS/MS (Waters Acquit Class I HPLC—ABSciex 5500 Q-Trap MS; Accucore Phenyl Hexyl Column, 2.1 mm × 100 mm × 2.6 µm; BetaSil C18 Javelin Guard Column, 2.1 mm × 20 mm × 5 µm) Waters Corporation. Milford, Massachusetts, United States (A: 2 mM ammonium formate in water with 0.1% formic acid, B: 2 mM ammonium formate in MeOH:ACN 1:1)	(No LOD/1–3) ng/mL	[73]
THC (Δ9) CBN CBD	Hair (Human)	Washed with 15 mL water, 10 mL acetone, and 10 mL hexane (2 min each). Dried at room temperature (2 h) and cut with scissors. 1.4 mL MeOH and 0.1 mL IS added to 20 mg of pieces.	HPLC-MS/MS (Shimadzu Prominence HPLC System—QTRAP 5500 MS; Kinetex C18 Column (2.1 mm × 100 mm × 1.7 µm) Shimadzu Corporation. Kyoto, Japan (A: Water containing 1 mM ammonium formate and 0.1% formic acid, B: ACN containing 1 mM ammonium formate and 1 mM formic acid)	(10/20) pg/mg	[74]
THC OH-THC THC-COOH CBN CBD	Hair (Human)	Washed 2× with MeOH. Cut in 1 mm pieces; 20 mg of hair hydrolyzed with 500 µL 1 M NaOH and 500 µL MeOH. Spiked with IS; 50 µL of acetic acid added. 4 mL of hexane/ethyl acetate added (90/10, *v*/*v*).	LC-MS (ExionLC AC Liquid Chromatograph—Sciex QTrap 6500+ MS; SCIEX. Framingham, Massachusetts, United States; Waters Acquity UPLC HSS T3 Column, 2.1 mm × 100 mm × 1.8 µm) Waters Corporation. Milford, Massachusetts, United States (A: Water with 2 mM ammonium formate and 0.1% formic acid, B: MeOH with 2 mM ammonium formate and 0.1% formic acid)	(0.2–10/ 0.2–50) ng/mg	[75]

SPE: Solid-phase extraction, SPP: Simple protein precipitation, LLE: Liquid-liquid extraction, SLE: Supported liquid extraction, ACN: Acetonitrile, IS: Internal Standard, LC-MS/MS: Liquid Chromatograph-Tandem Mass Spectrometer, UHPLC: Ultra High Performance Liquid Chromatography, HPLC: High Performance Liquid Chromatography, DI: Deionized, LLOQ: Lower Limit of Quantification, ULOQ: Upper Limit of Quantification.

**Table 3:** Sample Treatment Outline for Various Water Sample Types.

Method	Type	IS	Sorbent/Procedure **	Storage	Ref.
MSPE	WW	N/A	Fe_3_O_4_@poly(GMA/DVB-WAX) (30 mg) removed with magnet. Eluted with 2 mL 8% HCOOH/MeOH twice (4 mL total)	Polyethylene terephthalate bottles at −80 °C	[77]
SPE		Mixed standard solution of analytes at 200 ng/L in 200 mL ultrapure water	Oasis MCX and Oasis HLB Cartridges.		
SPE	WW SW DW	100 µL of 250 ng/mL analyte mix to 250 mL of either WW or SW OR 1 L of DW	Strata-X 33U Polymeric Reversed Phase (200 mg/6 mL). Conditioned with 6 mL MeOH and 6 mL distilled water. Eluted with 6 mL MeOH.	Polypropylene bottles at 4 °C	[46]
LLE	WW	N/A	50 µL working mix IS solution (200 µg/L) added to 25 mL of WW. NaCl added, 400 µL 1 M HCl added. 10 mL HX:EA (2:1) added.	Bottle material not specified. Stored at −20 °C	[25]
SLE		N/A	50 µL working mix IS solution (200 µg/L) added to 25 mL of WW.		
SPE		100 and 800 ng/L of IS mixture in 25 mL of Milli-Q water	Oasis HLB Cartridges (3 mL, 60 mg). Dilute with 75 mL Milli-Q water, add 50 µL working mix IS solution (200 µg/L). Condition cartridges with 6 mL MeOH and 6 mL Milli-Q water. Eluted with 5 mL MeOH.		
SPE	WW SW DW	50 ng/L of IS mixture in 200 mL water (1% MeOH)	Oasis HLB Cartridges (6 mL, 200 mg). Washed with 3 mL water, dried with nitrogen (5 min.) Eluted with 8 mL MeOH.	Dark bottles at 4 °C	[26]
Online SPE	WW SW	20 ng/L of IS mixture	Oasis HLB cartridges and Prospekt-2 Processor.	Bottle material not specified. Stored in the dark at −20 °C.	[42]
SPE	DW	N/A.	Polymeric cartridges (PLRP-s).	Amber polyethylene terephthalate bottles at −20 °C	[60]
SPE	WW	IS mixture in 200 mL effluent or 100 mL influent	Oasis HLB Cartridges. Eluted with MeOH.	Polyethylene bottles in the dark at −18 °C.	[50]
SPE	WW SW	N/A.	Oasis HLB Cartridges (3 mL, 60 mg). Conditioned with 3 mL MeOH and 3 mL Milli-Q water. Eluted with 5 mL MeOH.	Bottle material not specified. Stored at −20 °C	[39]
LLE	WW Biosolids (Sludge)	200 µL of IS mixture (200 ng/mL of cannabinoids + 20 ng/mL of synthetic cannabinoids)	20 mL of ultra-pure water and about 300 µL of 10% HCl added. 4 g of NaCl and 10 mL of ethyl acetate added. Centrifuged at 3220 RCF (no RPM provided) using Eppendorf, Model 5810, for 5 min to reduce emulsion. Evaporated at 45 °C using nitrogen.	Stored at −20 °C	[44]
SPE	WW SW	N/A	Oasis HLB, Oasis MAX, and Oasis MCX cartridges used. Conditioned with 6 mL MeOH and 6 mL water. Eluted with 6 mL.	Bottle material not specified. Stored at −20 °C	[40]
Online SPE	WW	IS mixture (200 ng/L)	Thermo Equan MAX Online SPE System. Conditioned with water, MeOH, and 1% formic acid/100 mM ammonium formate buffer.	Clear polyethylene terephthalate bottles in the dark at −20 °C	[43]
--	WW During Chlorination	0.1 mg/L of analyte (0.29 µM)	N/A	Bottle material not specified. Stored at 4 °C	[41]

MSPE: Magnetic solid phase extraction, SPE: Solid phase extraction, LLE: Liquid-liquid extraction, SLE: Solid-liquid extraction, WW: Wastewater, SW: Surface water, DW: Drinking water, IS: Internal standard. ** Procedure for SPE processes only go up to elution; the evaporation and reconstruction portion is not written. LLE only goes up to what is added.

**Table 4:** Sample Analysis Outline for Various Water Matrices Using LC-MS.

Analyte	Matrix	Instrument	Column	Mobile Phase	MS Mode	LOD/LOQ (ng/L)	Recovery (%)	Ref.
THC CBD CBD THC-COOH	WW	UHPLC-MS/MS	Waters Acquity UPLC HSS T3 (2.1 mm × 150 mm × 1.7 µm) Waters Corporation. Milford, Massachusetts, United States	A: 0.05% formic acid/2 mM ammonium formate/H_2_O B: 0.05% formic acid/acetonitrile	ESI (+), MRM	0.17–0.33/ 0.50–1.00	94.3–96.2	[77]
THC THC-COOH	WW (Influent/Effluent), SW, DW	UHPLC-MS/MS (1260 Infinity UHPLC—Agilent Technologies 6410 Triple Quad MS) Agilent Technologies. Santa Clara, California, United States	Sunfire C18 (2.1 mm × 50 mm × 3.5 µm)	A: MeOH/1 mM ammonium fluoride B: Water/1 mM ammonium fluoride Additives: 5 Mm ammonium formate, 5 mM ammonium acetate, 0.1% formic acid	ESI (−), NI mode	No LOD THC LOQ: WW: 1/0.5 SW: 0.3 DW: 0.1 THC-COOH LOQ: WW: 1.5/1 SW: 0.5 DW: 0.1	THC: 44–63% THC-COOH: 52–64%	[46]
THC OH-THC THC-COOH	WW (Influent)	UHPLC-MS/MS (Waters Acquity H- Class UPLC System—Xevo TQS Waters Triple Quad MS) Waters Corporation. Milford, Massachusetts, United States	Waters Acquity UPLC BEH C18 (2.1 mm × 50 mm × 1.7 µm) Waters Corporation. Milford, Massachusetts, United States	A: Milli-Q water/0.01% HCOOH and 5 mM NH_4_Ac B: MeOH	ESI (+)	1–3/ 3–10	62–80%	[25]
THC THC-COOH	WW (Influent/ Effluent), SW, DW (Raw)	UPLC-MS/MS	Waters Acquity UPLC BEH C18 (2.1 mm × 100 mm × 1.7 µm) Waters Corporation. Milford, Massachusetts, United States	A: MeOH B: 50 mM ammonium formate/formic acid buffer (pH 3.8)	ESI (+), SRM	THC: 2.3/ WW: 8.3 SW: 7.0 DW: <LOD-12.0 THC-COOH: 3.0/ WW: 12.5 SW: 10.0 DW: <LOD-14.7	N/A	[26]
THC THC-COOH	WW (Influent/ Effluent), SW	LC-ESI-MS/MS (Agilent HP 1100–4000 QTRAP Hybrid Triple Quad-Linear Ion Trap MS) Agilent Technologies. Santa Clara, California, United States	Purospher Star RP-18 End-Capped Column (2.0 mm × 125 mm × 5.0 µm)	Gradient Acetonitrile/Water	NI mode, SRM	N/A	N/A	[42]
THC THC-COOH OH-THC CBN CBD	DW	LC-MS/MS (Symbiosis-Pico System—4000 QTRAP Hybrid Triple Quad Linear Ion Trap MS)	Purospher Star RP-18 End-Capped Column (2.0 mm × 125 mm × 5.0 µm)	N/A	SRM	0.94–2.96/ 2.44–7.88	N/A	[60]
THC-COOH	WW (Influent/ Effluent)	LC-HRMS (Linear Ion Trap FT Orbitrap System)	XBridge C18	A: Water/0.05% Formic Acid B: MeOH/0.05% Formic Acid	DDA	N/A	N/A	[50]
THC-COOH TP	WW (Influent/ Effluent), SW	UHPLC-ESI-QqQ/QTOF MS (Waters Acquity UHPLC–Hybrid Quad Ortho Acceleration Time of Flight MS—Ortho Z-Spray ESI Interface)	Acquity UPLC BEH C18 (2.1 mm × 100 mm × 1.7 µm)	A: Water/0.01% Formic Acid B: MeOH/0.01% Formic Acid	ESI (−/+)	N/A	N/A	[39]
^A^ CBD CBD-COOH CBD-OH THC THC-COOH	WW Biosolids (Sludge)	LC-MS/MS (Sciex Exion LC—Sciex 6500+ QTrap—TurboSpray IonDrive) SCIEX. Framingham, Massachusetts, United States	Phenomenex Kinetix Biphenyl Column (2.1 mm × 50 mm × 1.7 µm)	A: Ultra-pure Water/MeOH (95:5) +0.01% Formic Acid B: MeOH/Ultra-pure Water (95:5) +0.01% Formic Acid	ESI (+), MRM	2.5–25/ 5–1000	Depends—multiple conditions tested for recovery.	[44]
^B^ THC THC-COOH CBN CBD CBG	WW (Effluent), SW	UHPLC-ESI-MS/MS (Waters Acquity H- Class UPLC System—Xevo TQS Waters Triple Quad MS) Waters Corporation. Milford, Massachusetts, United States	Acquity UPLC BEH C18 (2.1 mm × 100 mm × 1.7 µm)	A: MeOH B: Water	ESI (−/+), MRM	Reported in differing units/not one set unit for matrices and LOD/LOQ.	WW: 64–133 SW: 73–112	[40]
THC THC-COOH	WW (Influent)	HPLC-HRMS (Q Exactive Hybrid Quad-Orbitrap HRMS)	HypersSep Retain PEP (2.1 mm × 20 mm × 12 µm) and Hypersil Gold aQ Guard Column (2.1 mm × 10 mm × 3 µm)	A: Water, B: MeOH, D: 1% formic acid/100 mM ammonium formate	HESI-II (+), SRM	0.6–1.3/ No LOQ	THC: 97–131 THC-COOH: 119–122	[43]
THC-COOH	WW during chlorination	LC-QTOF-MS (Agilent 1200 Series—Agilent 6520 Series QTOF Hybrid MS) Agilent Technologies. Santa Clara, California, United States	Synergi Fusion-RP Column (2.0 mm × 100 mm × 4 µm)	A: Milli-Q Water/5 mM ammonium acetate B: MeOH/5 mM ammonium acetate	Dual ESI (−/+), EDR	N/A	N/A	[41]

UHPLC-MS/MS: Ultra High-Performance Liquid Chromatograph-Tandem Mass Spectrometer, LC-ESI-MS/MS: Liquid Chromatography-Electrospray Ionization-Tandem Mass Spectrometer,. LC-MS/MS: Liquid Chromatograph-Tandem Mass Spectrometer, LC-HRMS: Liquid Chromatograph-High Resolution Mass Spectrometry, LC-ESI-QqQ MS: Liquid Chromatograph-Electrospray Ionization-Triple Quadrupole Mass Spectrometer, UHPLC-ESI-MS/MS: Ultra High-Performance Liquid Chromatograph-Electrospray Ionization-Tandem Mass Spectrometer, ESI: Electron Ionization Spray, MRM: Multiple Reaction Monitoring, NI: Negative Ionization, SRM: Selected Reaction Mode, SRM: Selected Reaction Monitoring, DDA: Data-Dependent-Acquisition, HESI: Heated Electrospray Ionization Source, QTOF: Quadruple Time-of-Flight, EDR: Extended-Dynamic Range, WW: Wastewater, SW: Surface water, DW: Drinking water. ^A^ 45 other cannabinoids are tested within this study; ^B^ 4 other cannabinoids are tested within this study.

## Data Availability

No new data were created or analyzed in this study. Data sharing is not applicable to this article.

## Abbreviations

The following abbreviations are used in this manuscript:

CBC	Cannabichromene
CBD	Cannabidiol
CBG	Cannabigerol
CBN	Cannabinol
-COOH	Carboxy
CYP	Cytochrome P450 Enzymes
ESI	Electrospray Ionization
FDA	Food and Drug Administration
GC-MS	Gas Chromatography-Mass Spectrometry
HPLC	High-Performance Liquid Chromatography
LC-MS	Liquid Chromatography-Mass Spectrometry
MS/MS	Tandem Mass Spectrometry
-OH	Hydroxy
SFC	Supercritical Fluid Chromatography
THC	Tetrahydrocannabinol
